# High levels of 27-hydroxycholesterol results in synaptic plasticity alterations in the hippocampus

**DOI:** 10.1038/s41598-021-83008-3

**Published:** 2021-02-12

**Authors:** Raul Loera-Valencia, Erika Vazquez-Juarez, Alberto Muñoz, Gorka Gerenu, Marta Gómez-Galán, Maria Lindskog, Javier DeFelipe, Angel Cedazo-Minguez, Paula Merino-Serrais

**Affiliations:** 1 Department of Neurobiology, Care Sciences and Society, Division of Neurogeriatrics, Center for Alzheimer Research, Karolinska Institutet, Stockholm, Sweden; 2 Instituto Cajal (CSIC), Avenida Doctor Arce 37, 28002 Madrid, Spain; 3 Laboratorio Cajal de Circuitos Corticales, Centro de Tecnología Biomédica, Universidad Politécnica de Madrid, Campus Montegancedo S/N, Pozuelo de Alarcón, 28223 Madrid, Spain; 4 Departamento de Biología Celular, Universidad Complutense, Madrid, Spain; 5 Biodonostia Health Research Institute, Neuroscience Area, Donostia-San Sebastián, Gipuzkoa, Spain; 6 Department of Physiology and Pharmacology, Section for Anesthesiology and Intensive Care Medicine, Karolinska Institutet, Stockholm, Sweden; 7 Centro de Investigación Biomédica en Red Sobre Enfermedades Neurodegenerativas (CIBERNED), ISCIII, Madrid, Spain

**Keywords:** Cell biology, Neuroscience

## Abstract

Alterations in brain cholesterol homeostasis in midlife are correlated with a higher risk of developing Alzheimer’s disease (AD). However, global cholesterol-lowering therapies have yielded mixed results when it comes to slowing down or preventing cognitive decline in AD. We used the transgenic mouse model Cyp27Tg, with systemically high levels of 27-hydroxycholesterol (27-OH) to examine long-term potentiation (LTP) in the hippocampal CA1 region, combined with dendritic spine reconstruction of CA1 pyramidal neurons to detect morphological and functional synaptic alterations induced by 27-OH high levels. Our results show that elevated 27-OH levels lead to enhanced LTP in the Schaffer collateral-CA1 synapses. This increase is correlated with abnormally large dendritic spines in the *stratum radiatum*. Using immunohistochemistry for synaptopodin (actin-binding protein involved in the recruitment of the spine apparatus), we found a significantly higher density of synaptopodin-positive puncta in CA1 in Cyp27Tg mice. We hypothesize that high 27-OH levels alter synaptic potentiation and could lead to dysfunction of fine-tuned processing of information in hippocampal circuits resulting in cognitive impairment. We suggest that these alterations could be detrimental for synaptic function and cognition later in life, representing a potential mechanism by which hypercholesterolemia could lead to alterations in memory function in neurodegenerative diseases.

## Introduction

Alterations in brain cholesterol homeostasis have been correlated to neurodegeneration in numerous studies^1–5^. In particular, hypercholesterolemia in mid-life is considered as the main non-genetic risk factor for developing Alzheimer’s disease (AD) later in life^6–10^. Despite numerous discoveries in AD pathophysiology, the identification of strategies that can modify the onset and progression of AD remains a challenge, and therapies aiming to lower cholesterol in AD patients have not resulted in clear improvements in cognition^11^. Moreover, the mechanisms triggered by brain cholesterol homeostasis alterations that drive neurodegeneration remain unclear. In the brain, cholesterol is synthesized entirely in situ by astrocytes, and peripheral cholesterol does not cross the blood–brain barrier (BBB) under physiological conditions^12^. However, oxysterols, which are oxidized cholesterol metabolites, can cross the BBB by diffusion and influence brain cholesterol synthesis^12–16^.

27-hydroxycholesterol (27-OH), which is the most common oxysterol in the body, is produced in the periphery by the enzyme sterol 27-hydroxylase (Cyp27A1) and has a net influx into the brain driven by a concentration gradient^14,17^. Since 27-OH levels in the body are proportional to the cholesterol content in the blood, hypercholesterolemia leads to high 27-OH levels in the brain, where it can mediate negative effects on neuronal physiology^11,18–21^. We recently described that, in young Cyp27Tg mice (Cyp27A1-overexpressing mouse model), high levels of 27-OH downregulate the postsynaptic protein PSD-95 levels in the hippocampus and cause morphological alterations in neuronal structure and dendritic spine (for simplicity, spine) density in CA1 pyramidal neurons^22^.

Spines of pyramidal cells, the most common cell type in the cerebral cortex (including the hippocampus), are the main postsynaptic targets of excitatory synapses and their function is crucial for memory, learning, and cognition^23–25^. It is generally assumed that one spine establishes one excitatory glutamatergic synapse^26^. Therefore, the number of spines indicates the number of excitatory inputs that pyramidal cells receive. Moreover, it has been shown that spine volume correlates with the postsynaptic density area, as well as the number of presynaptic vesicles, and postsynaptic receptors^27–29^. Also, the length of the spine neck is related to its biochemical and electrical properties^30,31^. Consequently, alterations in spine density and morphology could alter the neuronal connectivity and induce circuit malfunction. Besides, a large number of alterations in spine microanatomy have been linked to AD and other neurodegenerative diseases^32–36^.

To test the functional effects of elevated 27-OH levels on hippocampal synaptic plasticity, we used a paradigm of long-term potentiation (LTP) in the CA1 region of the hippocampus in young Cyp27Tg mice. We found that elevated levels of 27-OH led to an increase in the level of hippocampal LTP, as evaluated by the field excitatory postsynaptic potentials (fEPSPs). Using intracellular injections with Lucifer Yellow (LY) and confocal microscopy, we reconstructed 3D spines in both, basal (*stratum oriens*) and apical (*stratum radiatum*) dendrites from CA1 pyramidal neurons. Our results show that spines from the apical dendritic tree are larger in Cyp27Tg compared with wild-type (WT) mice, but no changes were found in the *stratum oriens*. Although we did not find changes in glutamatergic receptor levels in the hippocampus, we found increased levels of synaptopodin puncta in the *stratum radiatum* of Cyp27Tg mice by immunohistochemistry. Collectively, our results show elevated levels of 27-OH in the brain affecting LTP, based on the alterations of the size of spines and their synaptopodin content at the early stages of postnatal development.

## Results

### 27-OH increases LTP in Cyp27Tg mice

**Figure 1:**
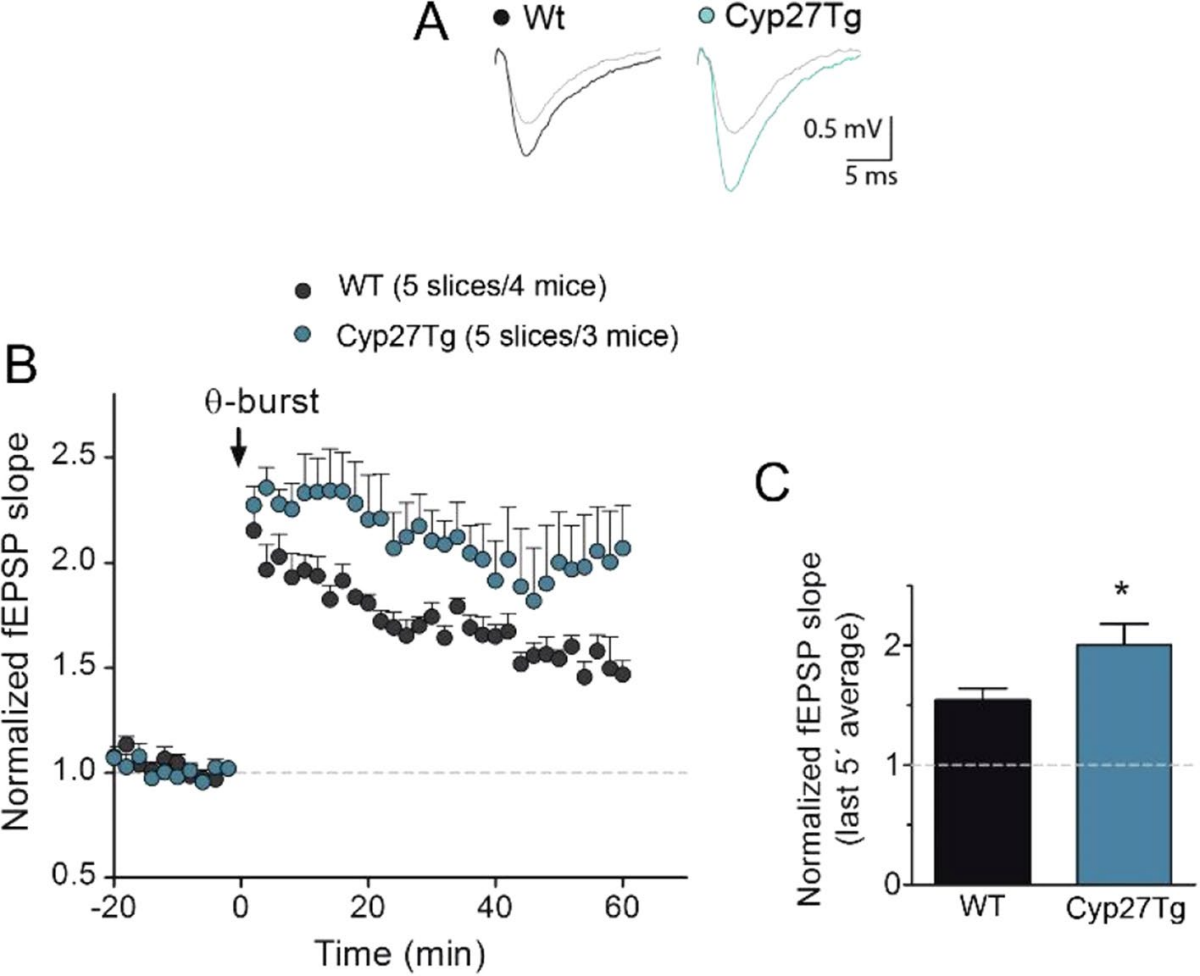
Altered Shaffer collateral-CA1 Long-term Potentiation (LTP) in Cyp27Tg mice. (**A**) Representative fEPSP traces before Ø-burst (gray traces) and 60 min after (black and turquoise traces). (**B**) Time-course of changes in field EPSP (fEPSP) slopes after induction of LTP show an increase in the magnitude of LTP induced by theta-burst (Ø-burst) stimulation in Cyp27Tg mice compared to WT hippocampal mouse slices. (**C**) Cyp27Tg mice (n = 5 slices/4 mice) showed significantly enhanced LTP compared to WT (n = 5 slices/3 mice) measured 55 to 60 min after Ø-burst relative to baseline; *p = 0.03 by unpaired Mann–Whitney test. Data are represented as the mean ± S.E.M.

To determine the effect of 27-OH overexpression on synaptic plasticity, we measured LTP in the CA1 region of the hippocampus of Cyp27Tg mice. For this purpose, evoked postsynaptic excitatory potentials were monitored through extracellular field recordings in the Schaffer collateral (SC)–CA1 pyramidal pathway. Stable evoked baseline activity was recorded for 30 min prior to the delivery of a theta-burst (Ø-burst) stimulation to induce LTP (Fig. 1). Cyp27Tg hippocampal slices showed a significantly higher magnitude of LTP when compared to hippocampal slices from WT animals (Fig. 1C; Cyp27Tg: 2.01 ± 0.17, n = 5 vs. WT: 1.54 ± 0.09, n = 5; *p = 0.03; unpaired Mann–Whitney test). In addition, Cohen's *d* was calculated to show the effect size (*d* = 1.47). The post-tetanic potentiation, measured as the average response during the first 5 min after delivering the stimulation, was not significantly different between the Cyp27Tg and WT mice (Cyp27Tg: 2.3 ± 0.07, n = 5; p = 0.16 vs. WT: 2.05 ± 0.11, n = 5; unpaired Mann–Whitney test). Additionally, the analysis of the input–output relationships recorded prior to LTP recordings showed no significant differences in basal synaptic transmission between WT and Cyp27Tg mice (Supplementary Fig. 1A; Two-way repeated measures ANOVA (F(1,8) = 0.87, p = 0.38). The assessment of the paired pulse ratio using 20, 40 or 60 ms of interstimulus interval showed no significant differences between genotypes at baseline (Two-way RM ANOVA F(1,4) = 0.0004,  p= 0.98) and LTP was not associated to changes in the paired pulse ratio at the tested interstimulus intervals in WT (Supplementary Fig. 1A, Two-way RM ANOVA F(1,2) = 0.06, p = 0.82) or Cyp27Tg mice (Two-way RM ANOVA F(1,2) = 4.11, p = 0.18). This result strongly suggests that the expression of LTP in the Cyp27Tg mice involves post-synaptic mechanisms similar to what is observed in WT; however, further detailed electrophysiological characterization would be necessary to fully confirm this observation.

### High 27-OH does not modify expression levels of NMDARs and AMPARs (GluR1/GluA1 and GluR2/GluA2) in the hippocampus

**Figure 2:**
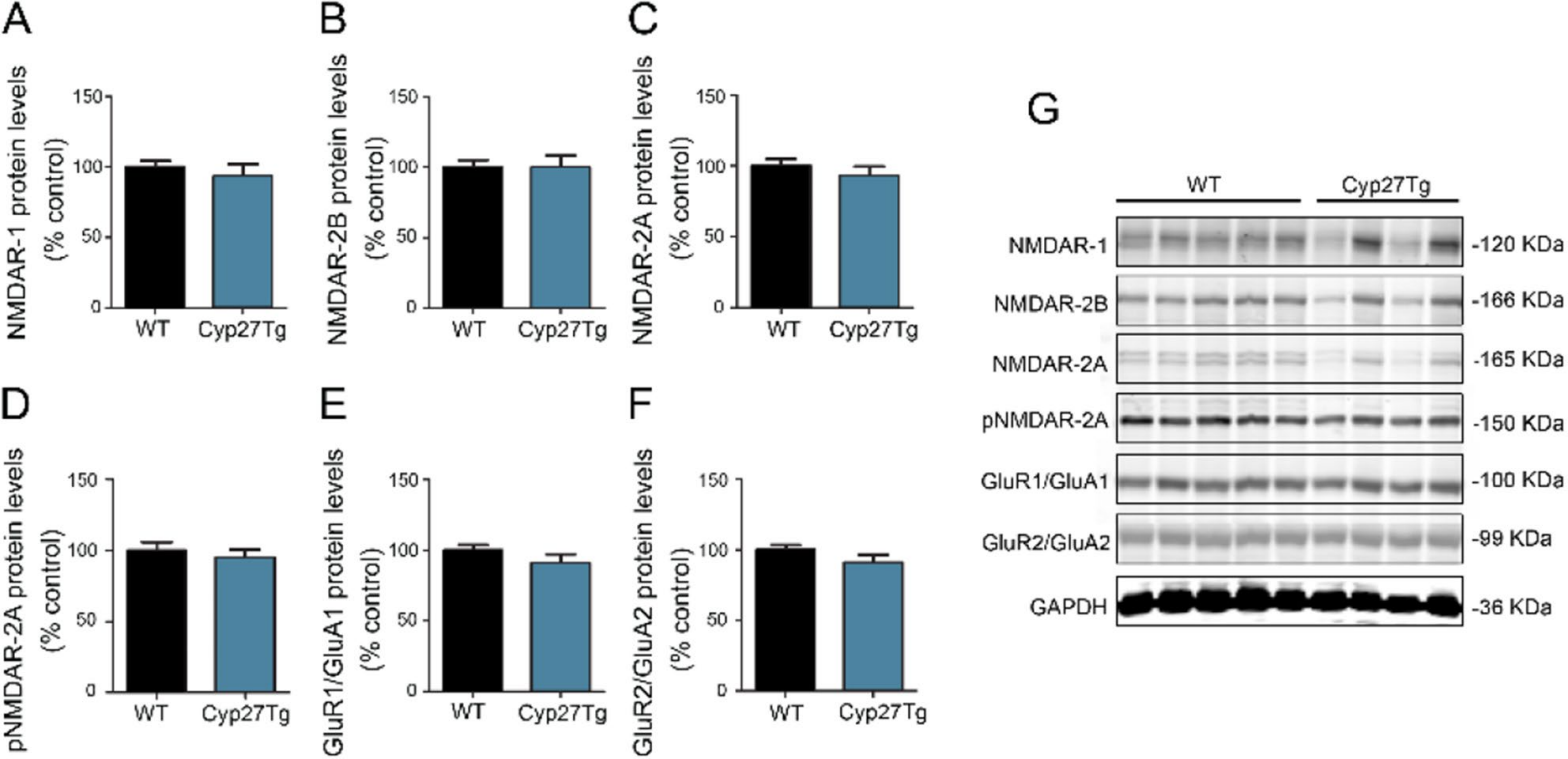
No changes in NMDARs and AMPARs hippocampal protein levels under high levels of 27-OH. (**G**) Western blot and (**A−F**) densitometry analysis showing the proteins levels (normalized to GAPDH) of (**A**) NMDAR-1, (**B**) NMDAR-2B, (**C**) NMDAR-2A, (**D**) pNMDAR-2A, (**E**) GluR1/GluA1 and (**F**) GluR2/GluA2 in the hippocampus of Cyp27Tg (n = 4) compared to WT mice (n = 5). Unpaired Mann–Whitney test was employed in each comparison to compare averages. All data are represented as mean ± SEM.

We performed immunoblotting analyses of the glutamate receptor N-methyl-D-aspartate receptor subunits (NMDARs) and receptor subunits 1 and 2 (GluR1/GluA1 and GluR2/GluA2) of the 2 α-amino-3-hydroxy-5-methyl-4-isoxazolepropionic acid receptors (AMPARs), in the hippocampus of Cyp27Tg and WT mice and no overt changes were found between groups (Fig. 2 and Supplementary Fig. 2). Cyp27Tg mice did not differ from WT mice regarding the levels of NMDAR-1 in the hippocampus (93.25 ± 8.61 vs. 100 ± 4.34, respectively) (p = 0.55; n = 4–5; unpaired Mann–Whitney test; Fig. 2A). Moreover, no differences were found between Cyp27Tg and WT mice when we compared protein levels for NMDAR-2B (Cyp27Tg, 100 ± 16.39; WT, 100 ± 11.26; p = 0.9; n = 4–5; unpaired Mann–Whitney test; Fig. 2B), NMDAR-2A (Cyp27Tg, 92.78 ± 6.67; WT, 100 ± 4.58; p = 0.55; n = 4–5; unpaired Mann–Whitney test; Fig. 2C) and phosphorylated NMDAR-2A (Cyp27Tg, 94.96 ± 5.48; WT, 100 ± 13.11; p = 0.73; n = 4–5; unpaired Mann–Whitney test; Fig. 2D) In parallel with possible differences in NMDARs hippocampal protein levels in Cyp27Tg compared to WT mice, we evaluated protein levels of AMPA-R. Expression levels of GluR1/GluA1 and GluR2/GluA2 subunits in the hippocampus were similar in Cyp27Tg and WT animals (GluR1/GluA1: Cyp27Tg; 90.89 ± 6.30; WT; 100 ± 3.67; p = 0.73, n = 4–5; unpaired Mann–Whitney test; Fig. 2E and GluR2/GluA2: Cyp27Tg; 90.90 ± 5.82; WT; 100 ± 3.48; p = 0.41; n = 4–5; unpaired Mann–Whitney test; Fig. 2F).

### Dendritic spines from CA1 pyramidal neurons are larger in the *stratum radiatum* in Cyp27Tg mice

**Figure 3:**
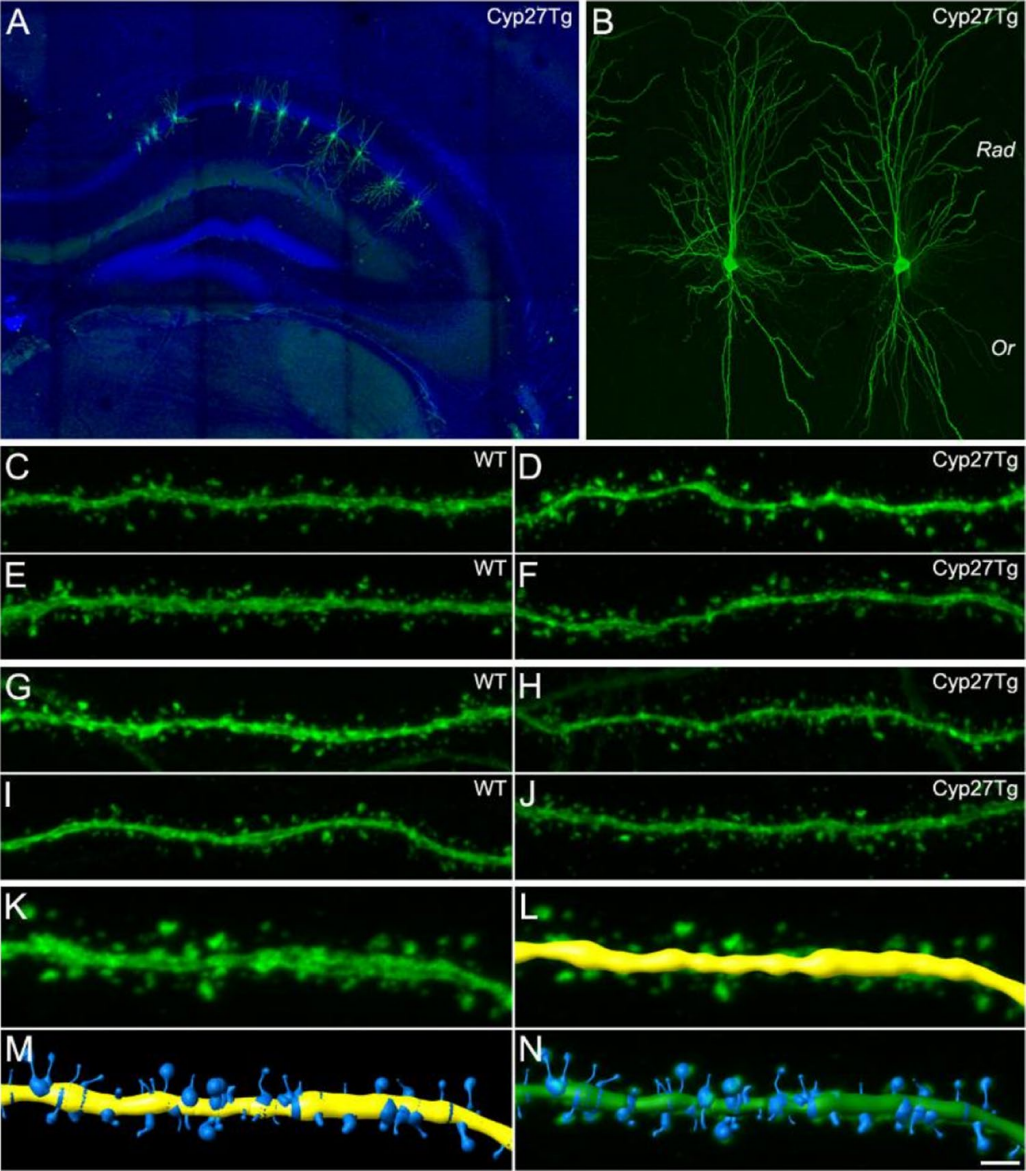
Intracellular injections and 3D spine reconstruction. (**A**) Hippocampal panoramic view taken from a Cyp27Tg mouse intracellularly injected in CA1 with LY (green) and stained with DAPI (blue). (**B**) Higher magnification of A showing single CA1 pyramidal neurons individually injected with LY. (**C−J**) Representative pictures (63X oil; pixel size 0.057 × 0.057; z-step 0.14) of basal (*Or*; *stratum oriens*) dendrites (**C−F**) and apical (*Rad*; *stratum radiatum*) dendrites (**G−J**) from WT (**C, E, G, I**) and Cyp27Tg (**D, F, H, J**) mice. (**K−N**) 3D reconstruction of the complete spine morphology. (**K**) Dendritic segment from a CA1 pyramidal neuron in the *stratum oriens* from a WT mouse. For each stack of images, a solid surface was created to match the dendritic shaft (**L**). Various intensity threshold surfaces were then created (**M**), and the solid surface that exactly matched the contours of the dendritic spine volume was then selected for each dendritic spine (**N**). Scale bar shown in N indicates 300 µm in **A, 40** µm in B, 10 µm in C−J and 5 µm in **K−N**.

To examine whether high levels of 27-OH could affect spine morphology from CA1 pyramidal neurons in Cyp27Tg mice, we performed a detailed 3D reconstruction analysis (Fig. 3). For this purpose, cells were labeled by intracellular injections in brain slices from 7- to 8-week-old WT and Cyp27Tg mice. A total of 182 pyramidal neurons from WT mice and 178 from Cyp27Tg mice were injected individually with LY in the CA1 region from the hippocampus (Fig. 3), and 3D images were computed by confocal microscopy. A detailed dendritic spine morphology analysis of the CA1 hippocampal subfield from Cyp27Tg mice was performed. Both basal dendrites (*stratum oriens*) and apical dendrites (*stratum radiatum*) were included in the analysis. For the statistical analysis, 4–5 animals per group were analyzed.

**Figure 4:**
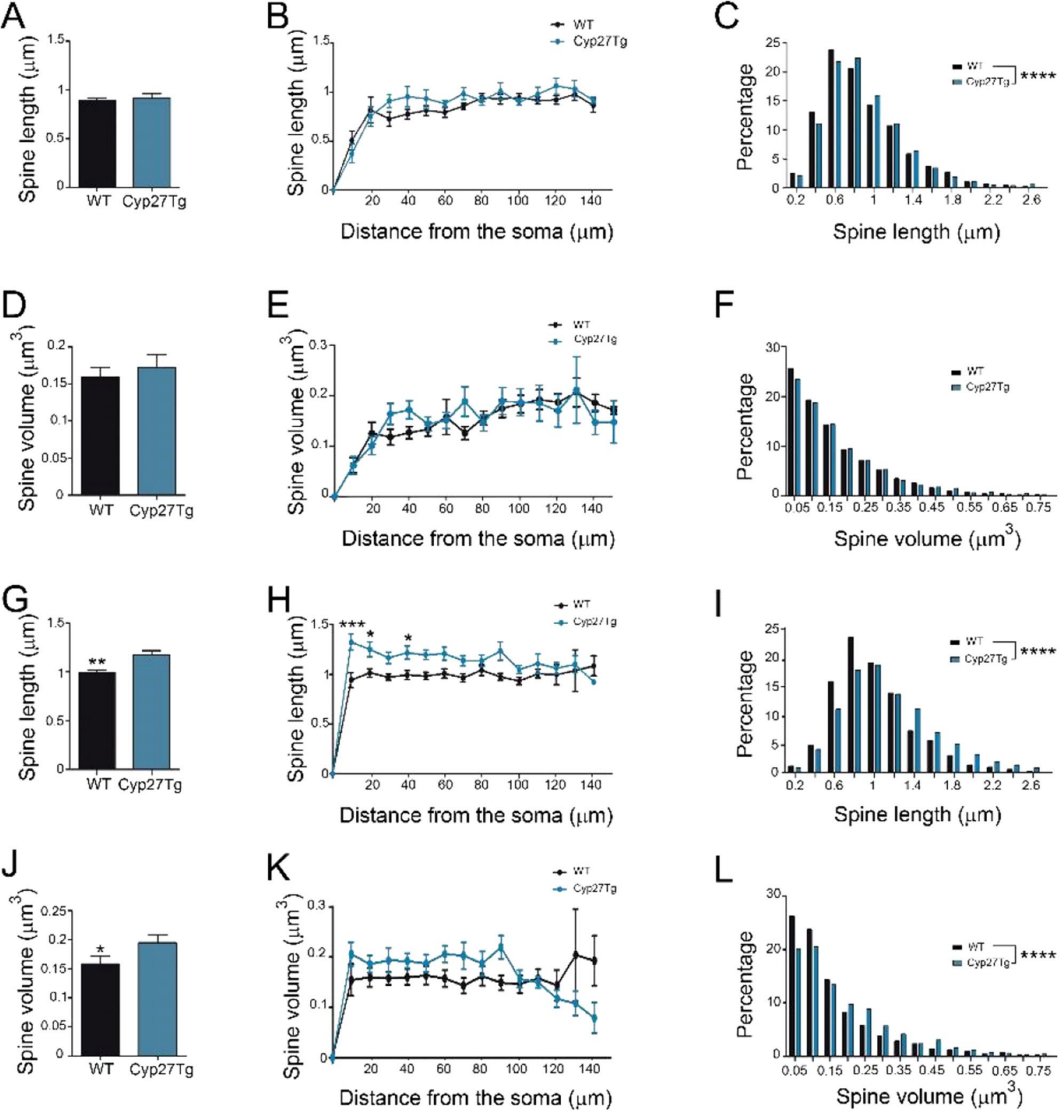
Dendritic spines from CA1 apical dendrites are larger in Cyp27Tg than in WT mice. Comparative morphometric analysis of spine length (**A**−**C**,** G**−**I**) and spine volume (**D−F,J−L**) for CA1 LY-injected pyramidal neurons in WT and Cyp27Tg mice for both basal (**A−F**) and apical (**G–L**) dendrites. Data are shown as average per dendrite (**A,D,G** and **J**; Unpaired Mann–Whitney test), as a function of the distance from the soma (**B,E,H** and **K**, Two-way ANOVA repeated measures followed by a post-hoc multiple Bonferroni test) and frequency distribution (**C,F,I** and **L**; Kolmogorov–Smirnov test). All data are represented as mean ± SEM. *p < 0.05; **p < 0.01; ***p < 0.001; ****p < 0.0001.

In the *stratum oriens*, spine morphology was measured by assessing a total of 4253 spines in WT mice and 2758 spines in Cyp27Tg mice. No statistically significant difference was found in the average spine length between Cyp27Tg (0.91 ± 0.04 μm; n = 19 dendrites) and WT mice (0.89 ± 0.02 μm; n = 25 dendrites; unpaired Mann Whitney test; p > 0.05; Fig. 4A). Likewise, when analyzing spine length as a function of the distance from the soma, no statistical significance was found (Two-way ANOVA, p > 0.05; F = 1.37; Fig. 4B). However, a significant difference was found between groups when we analyzed spine length using frequency distribution (Kolmogorov–Smirnov, p < 0.0001; Fig. 4C). Differences in spine volume did not show statistical significance between groups when it was analyzed as an average (WT, 0.15 ± 0.01 μm^3^; n = 25 dendrites; Cyp27Tg, 0.17 ± 0.07 μm^3^; n = 19 dendrites; unpaired Mann Whitney test; p > 0.05; Fig. 4D); as a function of the distance from the soma (Two-way ANOVA, p > 0.05; F = 2.37; Fig. 4E); or using frequency distribution (Kolmogorov–Smirnov, p > 0.05; Fig. 4F).

In the *stratum radiatum*, spine morphology was examined in apical branches protruding from the main apical trunk. These dendrites were located up to 300 µm from the *stratum pyramidale* (cell body layer). Dendrites located in the *stratum lacunosum-moleculare* were not included in the analysis. In the *stratum radiatum*, the spine morphology was measured by assessing a total of 2343 spines in WT mice and 2093 spines in Cyp27Tg mice. A significantly higher average dendritic spine length was found in Cyp27Tg (1.17 ± 0.04 μm; n = 20 dendrites) compared with WT mice (0.99 ± 0.02 μm; n = 20 dendrites; unpaired Mann Whitney test; p = 0.0028; Fig. 4G). Moreover, a statistically significant difference was found between groups regarding dendritic spine length as a function of the distance from the soma (Two-way ANOVA, p = 0.014; F = 2.56; p < 0.05, Bonferroni post-hoc test; Fig. 4H) and when using frequency distribution analysis (Kolmogorov–Smirnov, p < 0.0001; Fig. 4I). When we analyzed spine volume, a significantly higher average value was found in Cyp27Tg (0.19 ± 0.01 μm^3^; n = 20 dendrites) compared with WT mice (0.15 ± 0.01 μm^3^; n = 20 dendrites; unpaired Mann Whitney test; p = 0.04; Fig. 4J) and this was also the case when frequency distribution analysis was used (Kolmogorov–Smirnov, p < 0.0001; Fig. 4L). However, no statistical significance was found in the analysis of spine volume as a function of the distance from the soma (Two-way ANOVA, p > 0.05; F = 0.73; Fig. 4K).

### High 27-OH levels increase the number of synaptopodin immunoreactive puncta in *stratum radiatum*

**Figure 5:**
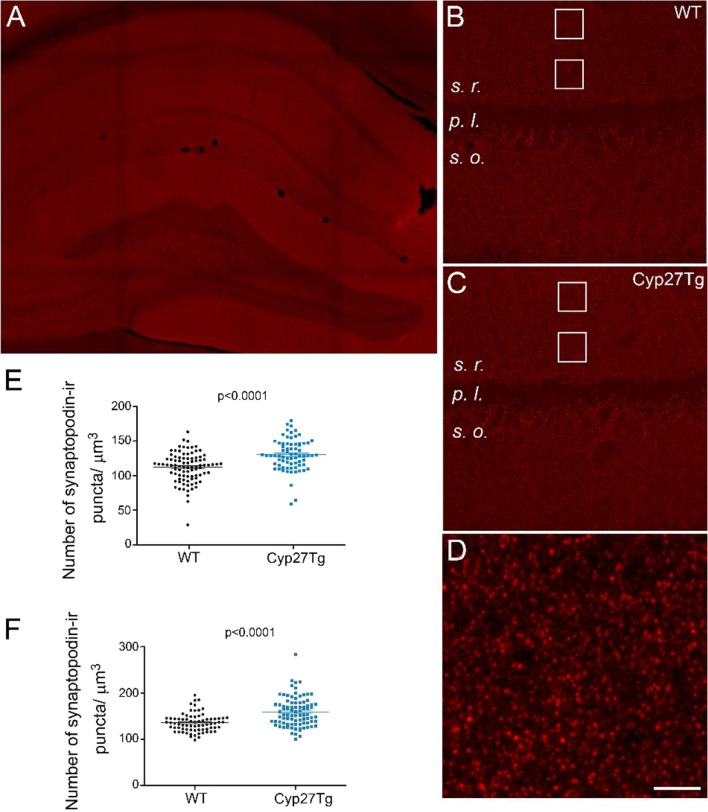
Cyp27Tg mice showed increased numbers of synaptopodin-ir puncta in the *stratum radiatum*. (**A**) Panoramic view of the hippocampus from a WT mouse stained with anti-synaptopodin antibody. (**B, C**) Representative pictures of the CA1 region in WT (**B**) and Cyp27Tg (**C**) mice, respectively. White squares represent the regions analyzed with confocal microscopy. Two different distances from the pyramidal layer were analyzed: 20−80 and 60−120 μm (*distal stratum radiatum*). (**D**) Photomicrograph in CA1 region from a WT mouse showing the synaptopodin-ir puncta distribution. (**E−F**) Comparative analysis showing scatter plots (GraphPad Prism 5.0) of the number of synaptopodin-ir puncta/ μm^3^ for the  (**E**) *stratum radiatum* and (**F**) *distal stratum radiatum* for Cyp27Tg compared to WT mice. Unpaired Mann–Whitney test was employed to compare groups. s.r., stratum radiatum; s.o., stratum oriens; p.l., pyramidal layer. All data are represented as mean ± SEM. Scale bar shown in A indicates 500 μm in A, 100 μm in B and C, and 10 μm in D.

The numbers of synaptopodin immunoreactive (ir) puncta were analyzed in the *stratum radiatum* from CA1 region in WT and Cyp27Tg mice (Fig. 5). This analysis was performed at two different distances from the pyramidal layer: 20–80 μm and 100–160 μm (Fig. 5B, C). In the *stratum radiatum*, we found a significantly higher number of synaptopodin-ir puncta in Cyp27Tg (132.1 ± 2.73 number of puncta/1000 μm^3^) than in WT mice (113.3 ± 2.32 number of puncta/1000 μm^3^; unpaired Mann Whitney test; p < 0.0001; n = 4–5 mice; Fig. 5E). In the *distal stratum radiatum*, 15% more synaptopodin-ir puncta were found in Cyp27Tg (159.5 ± 3.38 number of puncta/1000 μm^3^) than in WT mice (135.7 ± 2.3 number of puncta/1000 μm^3^; unpaired Mann Whitney test; p < 0.0001; n = 4–5 mice; Fig. 5F).

## Discussion

The present study aimed to test whether the alterations in neuronal morphology observed previously in CA1 of Cyp27Tg mice^22^ would correlate with synaptic plasticity dysfunction in the hippocampus. Our previous results show lower spine density in both apical and basal dendrites in Cyp27Tg compared with WT animals. However, the lower neuronal complexity was observed exclusively in the case of the basal dendritic tree in Cyp27Tg animals^22^. To address this aim, we performed LTP in brain slices of young adult Cyp27Tg mice and found an altered LTP response compared to WT mice. We found that LTP was increased in Cyp27Tg mice, with a steeper slope for the rise-time of fEPSPs 60 min after the theta-burst stimulation.

Our western blot results show that there was no difference in overall expression levels of NMDARs and AMPARs, and no increase in phosphorylation of the NMDAR-2A in Cyp27Tg mice compared to WT. Thus, neither the overall number of these receptors nor the sensitivity of the NMDA receptors^37,38^ seems to explain the increase in LTP. Thus, we decided to examine the microanatomy of the circuit.

Since the excitatory inputs that pyramidal cells receive are related to their number of spines, abundance and morphology^39–41^, we decided to analyze the morphological features of spines from both basal (*stratum oriens*) and apical (*stratum radiatum*) dendritic trees in CA1 pyramidal neurons. We found that globally, spines from apical dendrites were larger in Cyp27Tg mice than in WT counterparts. However, no differences were found in basal dendritic trees, suggesting that cortical circuits in the *stratum radiatum* and the *stratum oriens* might be affected differently by high levels of 27-OH. Distinct alterations in spine morphology between the CA1 *strata* in AD models have been previously described, suggesting this feature as a possible common hallmark in AD models^36^. Significant differences were found regarding spine volume and length, indicating that the spines from the apical dendritic tree, which receive input from the Schaffer collaterals, undergo both structural and functional plastic alterations.

Moreover, a detailed analysis of the morphological parameters as a function of the distance from the soma demonstrates that in apical dendrites the difference in spine length was only found up to 40 µm from the soma. It is well established that spine density and spine morphology along the dendrite is dependent on the distance from the cell body^42^, suggesting that synaptic input information processing varies depending on the location of the synaptic inputs on the dendrite. Since these plastic changes did not involve the upregulation of glutamate receptors, this led us to speculate whether the increase in volume and length of spines might be due to increased docking of glutamate receptors on the postsynaptic membrane.

Postsynaptic proteins are crucial structures of spines and are essential in memory acquisition and consolidation. In particular, PSD-95 and synaptopodin are important for learning and the plastic changes observed in LTP^43,44^ and also for synaptogenesis during development^45,46^. Previously, we reported lower PSD-95 levels in Cyp27Tg^22^; however, PSD-95 regulation is very dynamic^47^ and can be altered by the LTP paradigm^44^. Therefore, an enhancement in LTP response cannot be explained by PSD-95 downregulation. Moreover, previous work has reported an enhanced LTP response in the absence of PSD-95^48^. Synaptopodin, on the other hand, can control the expression and docking of glutamate receptors in response to several stimuli^49^. It can also affect the morphology of spines^50^ and is an essential structure of the LTP apparatus^51^. It has been shown previously that synaptopodin mRNA and/or protein expression is highly regulated under different conditions in podocytes and in various neuronal types^52–54^. Other mechanisms can alter the LTP response such as the GABAergic system^55^; however, synaptopodin expression is mostly restricted to excitatory pyramidal neurons^46^. In addition, behavioral experiments in old Cyp27Tg mice have not suggested any alterations in the GABAergic system^20^.

By immunostaining for synaptopodin in CA1, we found a significantly higher number of positive puncta in *stratum radiatum* of the pyramidal layer of Cyp27Tg compared to WT mice, which correlates with the microanatomical characterization discussed above. This implies that synaptopodin is specifically upregulated in spines in response to 27-OH leading to their enlargement. With larger spines in the apical dendritic tree, Cyp27Tg pyramidal neurons are more likely to potentiate the responses to glutamatergic input than WT are. Synaptopodin is an essential component of subpopulations of spines of hippocampal cells, in which it is critical for the recruitment of the spine apparatus which is involved in spine calcium kinetics and is required for the dynamic reorganization of the actin cytoskeleton in spine plasticity^46,51,56–58^.

Since 27-OH is present prenatally in Cyp27Tg mice, it is possible that regulatory mechanisms that are important for LTP have been depleted or abolished, as shown by the higher magnitude of the LTP response in these mice. In this regard, we found that elevated levels of 27-OH in the brain induce alterations in synaptic plasticity of CA1 pyramidal neurons, supported by upregulation of synaptopodin-positive puncta. We previously reported that high levels of 27-OH downregulate microRNA-124 (miR-124) expression in the hippocampus^22^ and other studies have found that the absence of miR-124 induces overexpression of synaptopodin in the spinal cord^59^. It is therefore, possible that the synaptopodin upregulation could be a consequence of the downregulation of miR-124 via an effect of 27-OH on spines, inducing an aberrant response to synaptic input from the Schaffer collaterals. An additional regulator of synaptopodin expression is aromatase, whose expression has been shown to decrease in response to calcium transients together with synaptopodin^60^. Therefore, it is possible that the effect of 27-OH on synaptopodin can be mediated by a cascade involving aromatase, although this correlation remains to be described. Moreover, other regulators of spine formation such as estrogen^61^ have been reported to be modulated by 27-OH^62^, however, the direct relationship of 27-OH and estrogen signaling in synaptogenesis is still uncharacterized. Similarly, the REST complex, also called RE1 silencing transcription factor, can lead to decrease dendritic spine size, as seen in genetic models of Down syndrome^63^. Since we have previously reported that 27-OH alters normal REST expression in primary neurons^22^, it is possible that REST can be another target linking 27-OH with dendritic spine alterations seen in this work.

We previously reported that high levels of 27-OH downregulate PSD-95 and decrease spine density in pyramidal neurons in the hippocampus^22^. We hypothesized that decreased PSD-95 levels would appear together with decreased glutamate receptors in the postsynaptic element and would alter the LTP function in the hippocampus. However, glutamate transporter levels were not significantly changed, whereas the LTP response was altered in Cyp7Tg mice. In the present study, we found that spine volume is increased, as well as the number of synaptopodin puncta in the *stratum radiatum* of the hippocampus. Overexpression of synaptopodin can lead to increased spine size and modify the CA1 LTP response^52,57^. Since synaptopodin is an essential regulator of the spine apparatus^64^, we propose that the specific upregulation of synaptopodin in spines in response to 27-OH may explain the spine enlargement. This could represent a compensatory mechanism in response to the decrease in PSD-95 and SHANK levels previously reported by our group^20^. Compared to WT, Cyp27Tg pyramidal neurons have larger spines in the apical dendritic tree and consequently are more likely to potentiate the responses to glutamatergic input. It is well established that LTP induces spine-remodeling and an increase in spine size^65–69^, but it is also clear that larger, mature spines can contain more of the signaling proteins necessary for LTP induction and may therefore be more easily potentiated^70,71^.

Consistent with this, we found that elevated levels of 27-OH in the brain induce increased LTP in CA1 pyramidal neurons. An enhanced LTP has previously been observed in other models for neurodegeneration such as AD^72^ and Nasu-Hakola disease^73^, which are characterized by alterations in cognition and learning. Although LTP at specific synapses is unequivocally associated with memory, a sustained increase in stimulation can lead to activation of cell death cascades^74^. However, increased activity and continuous release of glutamate have been shown to precede neurodegeneration^75–78^ and recent work suggests that an imbalance between homeostatic response and specific synaptic plasticity such as LTP may be an underlying cause of AD^79^. A lower LTP response is often seen in AD models^80^, but we must point out that not all AD models have reduced LTP. The APP_swe/ind_ mice, which have a double APP mutation, have increased LTP with clear signs of synaptic excitability and increased maximum amplitude of evoked EPSCs at 20 weeks of age^81^. The alterations in LTP observed in our model shows an aberrant response to the  high frequency stimulation of Schaffer collaterals in CA1, including an exacerbated sustained potentiaton of fEPSC. This is evidence of a deviation from the physiological LTP response, rather than an enhancement and it is most likely detrimental for hippocampal function, leading to the cognitive disorders seen in older Cyp27Tg mice^20^.

High 27-OH levels in the brain arise from a peripheral imbalance in cholesterol metabolism, which is related to non-genetic risk factors for neurodegeneration and cardiovascular disease^82,83^. In the present study, we found that despite the peripheral origin of 27-OH, its high levels can directly affect basic mechanisms of memory in the brain by altering the microanatomy of certain pyramidal neurons in the hippocampus and their response to synaptic input. Given its ubiquitous abundance in the brain, once it is elevated^84^, 27-OH will most likely affect multiple cell types and regions that undergo plasticity. Further research is required to fully understand all the detrimental mechanisms associated with elevated 27-OH in the central nervous system.

## Methods

### Animals

#### Cyp27Tg

We used a transgenic mouse model (Cyp27Tg) that overexpresses the enzyme Cyp27A1^21^. Since Cyp27A1 converts cholesterol to 27-OH^85^, Cyp27Tg mice have 5–6 times higher levels of 27-OH than control mice (WT) in serum (283 ± 11 vs. 48 ± 2 ng/ml) and in the brain (3.5 ± 0.5 vs. 0.3 ± 0.0 ng/mg) throughout life^20^, but have normal brain cholesterol levels^14^. In addition, Cyp27Tg showed a deficit in cognition^20^. Age-matched WT littermates served as controls. The mice were fed normal chow and water provided ad libitum and housed in groups of four or five with a 12 h light/dark cycle. Only two- to four-month-old males were used.

For immunoblotting analyses, an additional cohort of Cyp27Tg (n = 4) and WT mice (n = 5) were sacrificed by decapitation. The brains were dissected and immediately frozen on dry ice and stored at –80 °C until processing. An entire right hippocampus per animal was included in the western blot analysis. For intracellular injections and immunohistochemistry, the animals were overdosed with sodium pentobarbitone intraperitoneally. They were then perfused intracardially with 4% paraformaldehyde (PFA) and subsequently post-fixed in PFA for 24 h. The study was carry out in compliance with the ARRIVE guidelines.

#### Electrophysiology

Male mice (2 to 4 months old; 4 WT and 3 Cyp27Tg) were decapitated after been deeply anesthetized with isoflurane. Then, the brain was dissected out and placed in ice-cold standard artificial CSF (aCSF; 130 mM NaCl, 24 mM NaHCO_3_, 10 mM glucose, 1.25 mM NaH_2_PO_4_, 3.5 mM KCl, 1 mM MgCl_2_ and 2 mM CaCl_2_). 400 µm thick horizontal slices were prepared from the ventral hippocampus using a Leica VT1200S vibratome (Leica Microsystems). The slices were allowed to recover in an interface incubation chamber filled with 34 °C aCSF for 15–30 min and then left in the chamber that was allowed to cool down at room temperature. After a minimum recovery of 2 h, the slices were transferred to a submerged recording chamber with a perfusion rate of 2–3 ml per min with standard aCSF at 32 °C, constantly bubbled with carbogen gas (5% CO_2_, 95% O_2_).

LTP was examined in the Shaffer collaterals (SC)-CA1 pathway by evoking synaptic activity with an electrical stimulation using a bipolar concentric electrode (FHC Inc., USA) connected to an isolated current stimulator (Digitimer, UK) and recording field excitatory postsynaptic potentials (fEPSPs) using an extracellular recording pipette filled with regular aCSF. The recording electrode was placed around 50 to 100 µm from the pyramidal cell layer and the stimulation electrode was placed at the Schaffer collaterals at a distance of between 200 and 250 µm from the recording electrode. A stable fEPSP baseline response was collected by stimulation every 30 s for at least 30 min using 50–60% of the maximal response determined using a previously generated input–output curve. To induce LTP in the CA1 region, a protocol of theta-burst stimulation (Ø-burst) was used; a Ø-burst consisted of 2 trains with 10 bursts of 4 pulses at 100 Hz and bursts were delivered at 5 Hz. In some of the slices, a series of paired pulse facilitation experiments with inter-stimulus intervals of 10, 20 and 60 ms were performed after the baseline recording and 60 min after the induction of LTP. Responses were quantified by determining the slope of the linear rising phase of the fEPSP (between 10 and 70% before reaching the peak amplitude). The response was normalised to baseline response for each slice and the magnitude of LTP was determined by comparing the five-minute average of the fEPSP slope 60 min after the Ø-burst stimulation with the average of the last five minutes of the baseline recording.

### Intracellular injections, reconstruction and 3D morphometric analysis

Coronal whole brain sections (150 μm) were obtained using a vibratome. The sections were prelabeled with 4,6 diamino-2-phenylindole (DAPI; Sigma, St Louis, MO). CA1 pyramidal neurons were then individually injected with LY by continuous current as previously described^22^. Imaging was performed with a Nikon Ti-E inverted point scanning confocal system with a 488 nm Argon laser and UV (405 nm). The fluorescence of DAPI and Alexa 488 was recorded through separate channels. We obtained image stacks of 10–100 image planes with a 20 × dry lens (NA, 0.75). No pixels were saturated within the spines. After acquisition, the stacks were analyzed with 3D image processing software — Imaris 9.1 (Bitplane AG, Zurich, Switzerland) by a person blinded to the experimental conditions.

Morphometric parameters of spines: The volume and the length of the spines were established blindly by semiautomatic reconstruction using the Filament tool from Imaris.

For the morphological analysis, an optimal sampling of the dendrites was performed. We selected exclusively the dendrites that were homogeneously infused to avoid differences in LY intensities due to experimental procedures. Briefly, several filaments were created for each stack of images, and the parameters were modified until the contours matched with the volume of the spines. The image of each dendrite was rotated in 3D and examined to ensure that the selected contour for each spine was appropriate. In addition, the spine volume and spine length were measured as a function of the distance from the soma. Concentric spheres of increasing 10 μm radii were created (Sholl analysis).

### Immunoblotting analysis

Western blot analysis was carried out in tissue from the whole hippocampi as described previously^22^. 20 µg of total soluble protein from whole hippocampal lysates were loaded into acrylamide gels and separated using SDS-PAGE. After being transferred to a nitrocellulose membrane (Schleicher & Schuell, Germany), milk-blocked blots were incubated overnight with the following antibodies: anti-GluR1 (ab109450, rabbit monoclonal, 1:1000; Abcam, UK), anti-GluR2 (ab20673, rabbit polyclonal, 1:1000; Abcam, UK), anti-NMDAR1 (ab109182, rabbit monoclonal, 1:1000, Abcam, UK), anti-NMDAR-2A (ab124913, rabbit monoclonal, 1:1000; Abcam, UK), anti-phosphorylated-NMDAR-2A (Y1325, ab106590, rabbit polyclonal; Abcam, UK), anti-NMDAR-2B (ab28373, mouse monoclonal, 1:1000; Abcam, UK), and anti-GAPDH (ab8425, mouse polyclonal, 1:1000; Enzo, USA). Secondary incubation was carried out using anti-rabbit or anti-mouse IRDye infrared IgG antibodies (Li-Cor Biosciences, USA) at a 1:5000 dilution at room temperature. Immunoreactivity was detected using Odyssey CLx Imaging System (Li-Cor Biosciences, USA). The densitometric analyses of the immunoreactive bands were performed using Image Studio Lite ver. 5.2 (Li-Cor Biosciences, USA). Some immunoblots were stripped using Restore Western Blot Stripping buffer (Pierce, Rockford, IL, USA) at room temperature for 15 min and then blocked and re-blotted with other antibodies.

### Immunofluorescence and synaptopodin-positive puncta quantification analysis

Free-floating coronal Sects. (50 μm) were used for single immunofluorescence. First, the slices were blocked for 1 h in PB (0.1 M) with 0.25% Triton X -100 and 3% normal goat serum (Vector Laboratories Inc., Burlingame, CA, USA). Then, the sections were incubated overnight with the primary antibody rabbit anti-synaptopodin (SE-19; S9442; Sigma; 1:500) at 4 °C and subsequently incubated for 2 h at room temperature with Alexa Fluor 594-conjugated goat anti-rabbit antibody (A11012, Molecular Probes, Eugene, OR, 1:1000). Last, the sections were counterstained with DAPI (4, 6-diamidino-2-phenylindole; Sigma, San Louis; MO; 1:80), mounted and coverslipped with ProLong Gold antifade reagent for immunofluorescence analysis (Life Technologies, Carlsbad, CA). For synaptopodin immunostaining validation, double immunofluorescence was performed with synaptopodin (described above) and the presynaptic marker for the vesicular glutamate transporter 1 (VGLUT-1; AB5904, Millipore, Massachusetts, USA, 1:5000) antibodies (Supplementary Fig. 3). Free-floating sections were incubated overnight at 4 °C in a solution containing the primary antibodies, and then for 2 h at room temperature with biotinylated goat anti-guinea pig antibody for VGLUT-1 (1:200: Vector). After rinsing in PB, the sections were incubated for 2 h at room temperature with streptavidin coupled to Alexa fluor 488 (1:2,000, S-32354, Molecular Probes) and Alexa Fluor 594-conjugated goat anti-rabbit antibody. The sections were finally counterstained with DAPI, mounted and coverslipped with ProLong Gold antifade reagent for immunofluorescence analysis.

For synaptopodin-positive puncta quantification analysis, confocal microscopy was performed with a Zeiss LSM 710 confocal laser scanning system (Carl Zeiss Microscopy GmbH, Jena, Germany). The puncta were recorded with a 63 × objective (NA, 1.4) through separate channels (image resolution 1024 × 1024 pixels; Image size 58.67 μm × 58.67 μm). Z depth in every confocal stack was 0.14 μm. We scanned 9 image stacks from the *stratum radiatum* of the CA1 region from each section (two sections per animal). The stacks were taken at two different distances from the pyramidal layer: 20–80 μm and 100–160 μm (*distal stratum radiatum*). For the quantification of the synaptopodin-positive puncta, Image J software (a 3D object counting tool) was used. The image stacks were cropped to ensure that they were restricted to areas of fine neuropil. Synaptopodin fluorescence was automatically enhanced and then single-pixel background fluorescence was eliminated by de-speckling. The 3D object counting tool was then applied to collect data regarding the number and size of synaptopodin-ir punctate elements excluding those in contact with exclusion borders. To estimate density values, the number of synaptopodin-ir puncta was then normalized according to the tissue volume of each stack. To ensure impartiality, all the measurements were done blindly by the same investigator.

### Statistical analysis

To test the overall effect, unpaired Mann–Whitney test was used to compare the averages. For spine morphology analysis, Two-way ANOVA repeated measures (*P* and *F* values + interaction are shown) followed by a post-hoc multiple Bonferroni test were used to compare values as a function of the distance from the soma. Kolmogorov–Smirnov test was used to perform frequency distribution analysis. Data values are expressed as mean ± SEM. In all cases, p < 0.05 was considered to be significant (* < 0.05, ** < 0.01, *** < 0.001, **** < 0.0001).

In addition, Cohen's *d* was calculated to test the effect size in the electrophysiological analysis. Cohen's *d* was obtained as the difference between the means divided by the pooled SD of both groups at baseline. Cohen's *d* can define effect sizes that are small (*d* = 0.2 to 0.49), medium (*d* = 0.5 to 0.79), and large (*d* ≥ 0.8).

### Ethical approval

All experimental procedures were conducted following European relevant guidelines and regulations and were approved by the Swedish Board of Agriculture (ethical permits ID S33-13, extension 57-15 and 4884/2019).

## Supplementary Information

Supplementary Information.
