# Supplementary material for: High levels of 27-hydroxycholesterol results in synaptic plasticity alterations in the hippocampus
Source: Sci Rep. 2021 Feb 12;11:3736. doi: 10.1038/s41598-021-83008-3 (PMC7881004; doi:10.1038/s41598-021-83008-3)
Supplement: Supplementary file 1 — Supplementary Information. [file 41598_2021_83008_MOESM1_ESM.pdf]

High levels of 27-hydroxycholesterol results in synaptic plasticity alterations in the hippocampus

Raul Loera-Valencia[1], Erika Vazquez-Juarez[1], Alberto Muñoz[2,3,4], Gorka Gereñu-Lopetegui[1,5], Marta Gómez-Galán[6], Maria Lindskog[1], Javier DeFelipe[2,3,7], Angel Cedazo-Minguez[1*] and Paula Merino-Serrais[1,2,3*]

[1]Department of Neurobiology, Care Sciences and Society, Division of Neurogeriatrics, Center for Alzheimer Research, Karolinska Institutet, Stockholm, Sweden

[2]Instituto Cajal, CSIC, Madrid, Spain

[3]Laboratorio Cajal de Circuitos Corticales (CTB), Universidad Politécnica de Madrid, Madrid, Spain

[4]Departamento de Biología Celular, Universidad Complutense, Madrid, Spain

[5]Biodonostia Health Research Institute, Neuroscience Area, Donostia-San Sebastián, Gipuzkoa, Spain

[6]Department of Physiology and Pharmacology, Anestesiologi laboratory, Karolinska Institutet, Stockholm, Sweden

[7]Centro de Investigación Biomédica en Red sobre Enfermedades Neurodegenerativas (CIBERNED), ISCIII, Madrid, Spain

*Correspondence to:

Paula Merino-Serrais

Instituto Cajal (CSIC), Avenida Doctor Arce 37, 28002 Madrid, Spain; or

Laboratorio Cajal de Circuitos Corticales, Centro de Tecnología Biomédica,

Universidad Politécnica de Madrid, Campus Montegancedo S/N, Pozuelo de Alarcón, 28223 Madrid

E-mail: paula.merino-serrais@cajal.csic.es

and

Angel Cedazo-Minguez.

Center for Alzheimer Research, Division of Neurogeriatrics, Department of Neurobiology,

Care Sciences and Society, Karolinska Institutet, Stockholm

E-mail: angel.cedazo-minguez@ki.se

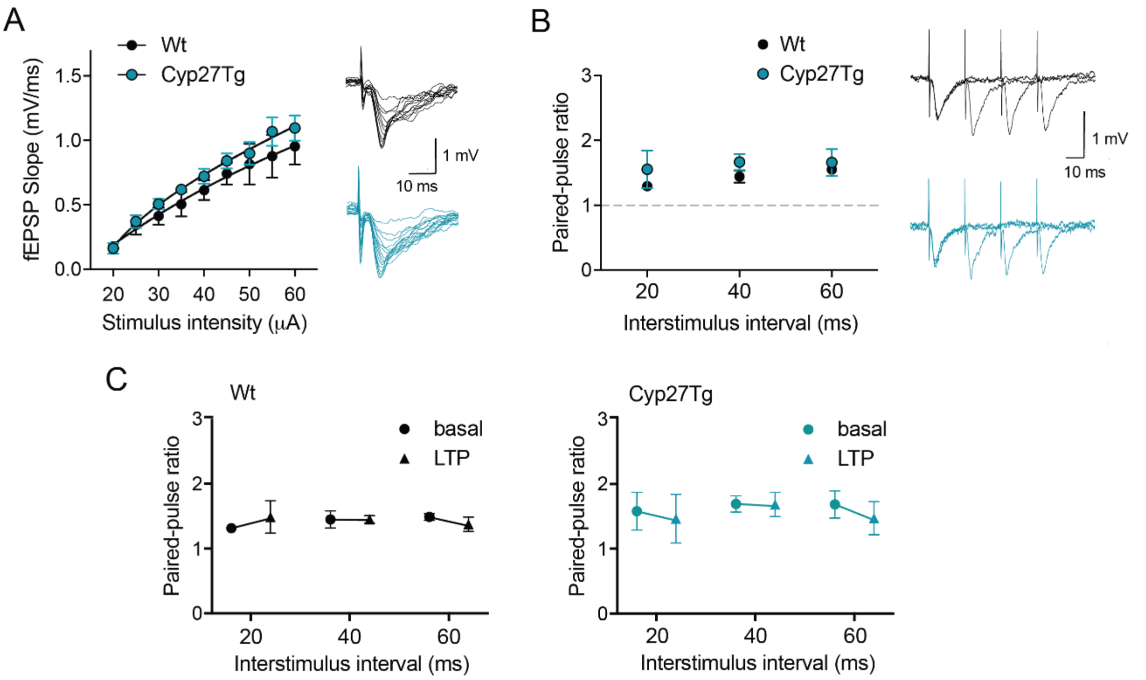

Supplementary Figure 1. Analysis of the input-output included in Figure 1. (A) Input-output relationships showing the fEPSP slope as a function of stimulus intensity. Two-way repeated measures ANOVA showed no significant difference between Wt and Cyp27Tg mice (F(1,8)=0.87, P=0.38). (B) Paired pulse facilitation expressed in terms of the ratio (fEPSP2/fEPSP1) at 20, 40 or 60 ms interstimulus intervals showed no significant differences between genotypes (Two-way RM ANOVA F(1,4) = 0.0004, P=0.98). (C) LTP did not further modified significantly the paired pulse ratio at the tested interstimulus intervals in Wt (Two-way RM ANOVA F(1,2) = 0.06, P=0.82) or Cyp27Tg mice (Two-way RM ANOVA F(1,2) = 4.11, P=0.18).

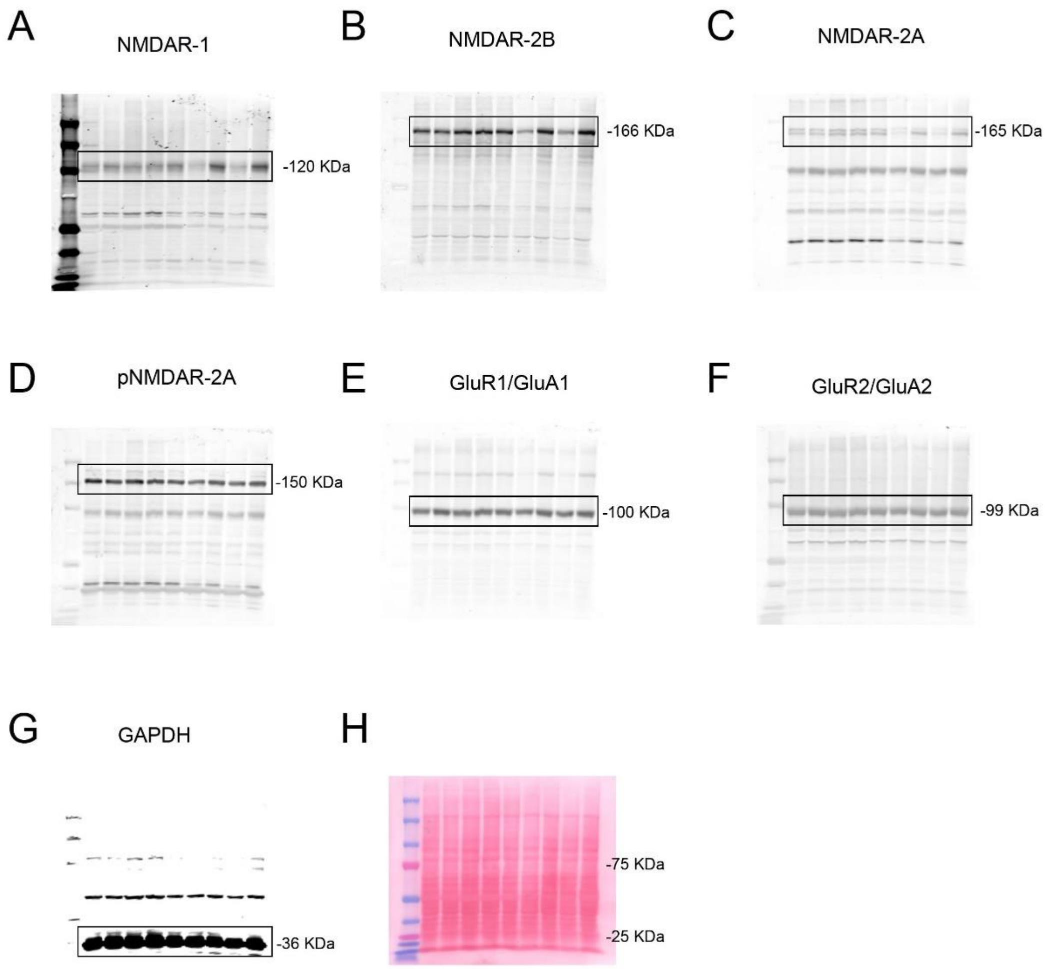

Supplementary Figure 2. Full-length images for the western blots included in Figure 2. Western blot analysis of the glutamate receptor N-methyl-D-aspartate receptor subunits: (**A**) NMDAR-1, (**B**) NMDAR-2B, (**C/D**) NMDAR-2A/pNMDAR-2A; and receptor subunits of the 2 α-amino-3-hydroxy-5-methyl-4-isoxazolepropionic acid receptors: (**E**) GluR1/GluA1and (**F**) GluR2/GluA2. (**G**) GAPDH was used as control and (**H**) Ponceau S staining as a quality control test. Black boxes indicate the cropped section as displayed in the main body figure.

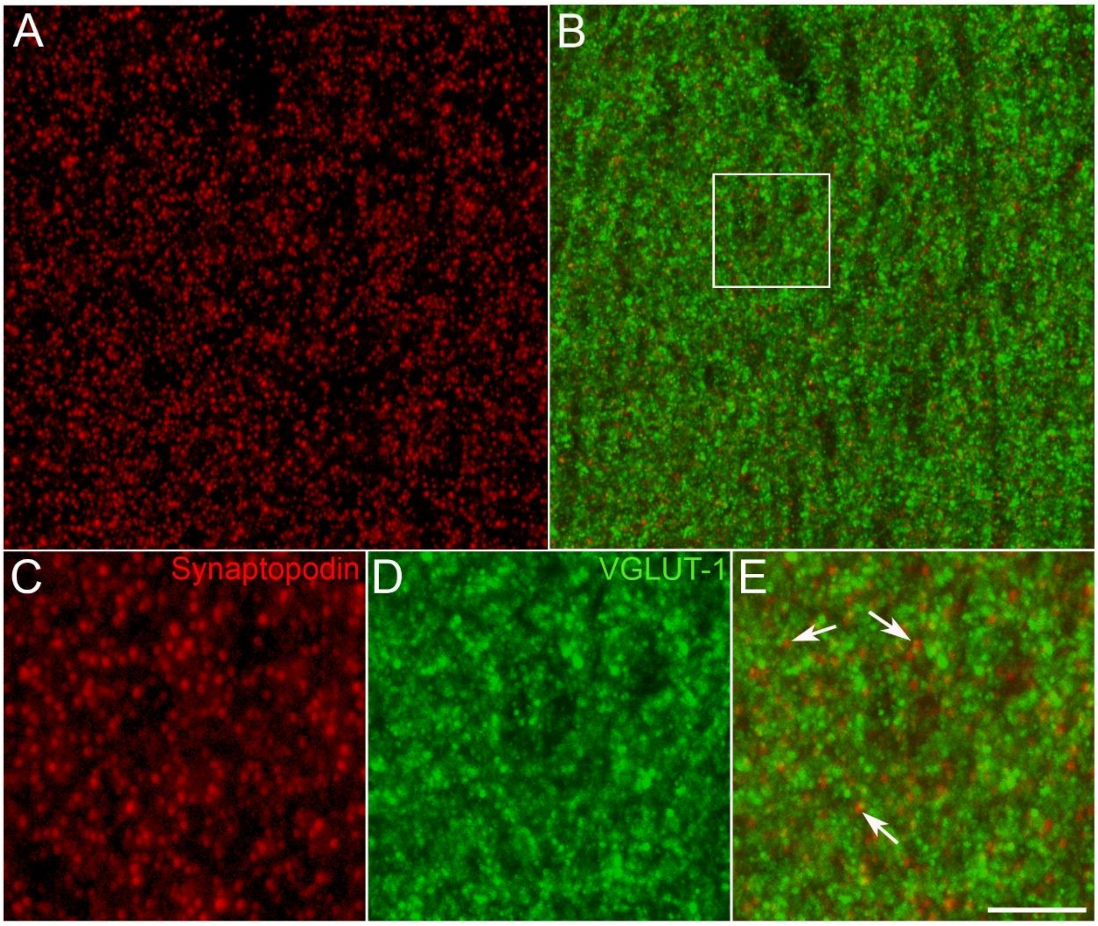

Supplementary Figure 3. Double immunofluoresce images using antibodies for synaptopodine and VGLUT-1 in the *stratum radiatum* of a WT mouse. (**A, C**) Confocal images (63x objective; NA, 1.4; image resolution 1024×1024 pixels) showing synaptopodin immunoreactive puncta to be quantify as shown in Figure 5. (**B, D**) Staining for the presynaptic marker VGLUT-1. (**E**) Merge of C and D. Note the lack of colocalization of immunoreactivities for synaptopodin and VGLUT-1. Arrows indicate some synaptopodin puncta adjacent to VGLUT-1 puncta. Scale bar shown in E indicates 15 µm in A- B and 5 µm in C-E.
